# Design of a Phenotypic Sensor About Protein and Moisture in Wheat Grain

**DOI:** 10.3389/fpls.2022.881560

**Published:** 2022-05-06

**Authors:** Yiming Liu, Donghang Li, Huaiming Li, Xiaoping Jiang, Yan Zhu, Weixing Cao, Jun Ni

**Affiliations:** ^1^College of Agriculture, Nanjing Agricultural University, Nanjing, China; ^2^National Engineering and Technology Center for Information Agriculture (NETCIA), Nanjing, China; ^3^Engineering Research Center of Smart Agriculture, Ministry of Education, Nanjing, China; ^4^Collaborative Innovation Center for Modern Crop Production Co-sponsored by Province and Ministry, Nanjing, China

**Keywords:** sensor, quality phenotype, near-infrared, multi-source circular structure, Fresnel reflectance concentrator, optical simulation, feedback driver, neural network modeling

## Abstract

A near-infrared (NIR) spectrometer can perceive the change in characteristics of the grain reflectance spectrum quickly and nondestructively, which can be used to determine grain quality information. The full-band spectral information of samples of multiple physical states can be measured using existing instruments, yet it is difficult for the full-band instrument to be widely used in grain quality detection due to its high price, large size, non-portability, and inability to directly output the grain quality information. Because of the above problems, a phenotypic sensor about grain quality was developed for wheat, and four wavelengths were chosen. The interference of noise signals such as ambient light was eliminated by the phenotypic sensor using the modulated light signal and closed sample pool, the shape and size of the incident light spot of the light source were determined according to the requirement for collecting the reflectance spectrum of the grain, and the luminous units of the light source with stable light intensity and balanced luminescence were developed. Moreover, the sensor extracted the reflectance spectrum information using a weak optical signal conditioning circuit, which improved the resolution of the reflectance signal. A grain quality prediction model was created based on the actual moisture and protein content of grain obtained through Physico-chemical analyses. The calibration test showed that the R^2^ of the relative diffuse reflectance (RDR) of all four wavelengths of the phenotypic sensor and the reflectance of the diffusion fabrics were higher than 0.99. In the noise level and repeatability tests, the standard deviations of the RDR of two types of wheat measured by the sensor were much lower than 1.0%, indicating that the sensor could accurately collect the RDR of wheat. In the calibration test, the root mean square errors (RMSE) of protein and moisture content of wheat in the Test set were 0.4866 and 0.2161%, the mean absolute errors (MAEs) were 0.6515 and 0.3078%, respectively. The results showed that the NIR phenotypic sensor about grain quality developed in this study could be used to collect the diffuse reflectance of grains and the moisture and protein content in real-time.

## Introduction

Real-time non-destructive testing of grain quality can lower the cost of field management by providing reference information for fertilization in the grain production process. It can also provide information for quality-based pricing during trade and provide data for grain classification to improve the efficiency of grain processing. However, traditional grain quality detection methods mainly rely on indoor Physico-chemical analysis. For instance, the grain moisture content was determined by the direct drying method (The National Health and Family Planning Commission of the People’s Republic of China, 2016), and the protein content was determined by the Kjeldahl method (The National Health and Family Planning Commission of the People’s Republic of China and State Food and Drug Administration, 2010). These methods cannot be applied to real-time quality detection in the process of grain circulation due to destructive sample preparation, complex analysis process, and poor timeliness (El-Mesery et al., 2019; Hussain et al., 2019).

Near-infrared (NIR) spectroscopy has developed rapidly as an efficient, green, and nondestructive analysis and detection method in recent years. The response characteristics of the NIR reflectance spectrum of grain are closely related to grain quality. The quality of grains can be described quantitatively using the NIR wavelength that is sensitive to the quality content (Wang, 2010; Santos et al., 2013; Zhu et al., 2015; Caporaso et al., 2018). By collecting the NIR transmitted spectrum of six types of single-grain wheat, a prediction model for NIR transmitted spectrum and the protein content of corresponding single-grain wheat was proposed by Delwiche (1995) based on partial least square. Using the Foss InfraXact^™^ Lab/Pro spectrometer (570–1850 nm), Arazuri et al. (2012) collected the spectrum of wheat in the NIR area, analyzed the relationships of rheological parameters between spectrum and tenacity, extensibility, deformation energy of wheat, which provided technical support for obtaining the rheological parameters of wheat during harvest and transportation of wheat. In a study by Li et al. (2013). a spectrometer was used to collect the transmitted spectrum (840–1,048 nm) of brown rice. This information was used to accurately determine the moisture and protein content of brown rice in the grain elevator. In summary, the above studies on NIR of grain showed that the quality information, e.g., moisture, starch, and protein content of grains (e.g., wheat and rice) can be obtained quantitatively.

Based on the NIR spectroscopy technology, grain quality detection devices have been developed. Zhang et al. designed a NIR analysis system with a wavelength of 800–1,100 nm based on a charge-coupled device and fixed optical grating, and a wheat moisture and protein model was created using the PLS method. However, this system was complex and large, it could not be used for real-time on-site detection (Zhang et al., 2009). Hidaka et al. developed a NIR reflectance spectrometer of 740–1,140 nm using the halogen lamp and grating dispersion, which was integrated with the harvester to determine the protein content of brown rice. However, when tested in the field, the correlation coefficient R of the detection value and the real value of protein was only 0.65 since the system did not take into account the influence of complex environment factors (Hidaka et al., 2011). In a study by Wen et al. light-emitting diodes (LEDs) and 14 narrow-band interference filters with a wavelength between 800 and 1,100 nm were used to develop a single-grain wheat composition analyzer, which realized the real-time detection of wheat protein content. However, the luminous efficiency of the LED of 950 nm was very low, at 1,020–1,050 nm, and the measurement error under repeated sample loading conditions was largely due to the light spot difference caused by the position of the light source (Wen and Ji, 2004). Wu et al. used a 6-row-8-column full-enclosed LED structure, combined with a FLAME-NIR spectrometer (900–1,700 nm) manufactured by Ocean Insight (Orlando, FL, United States), to design an instrument for detecting the protein content of single-grain wheat. This instrument requires a large number of light sources and has large power consumption, and it can only be used after the light source has been preheated for 30 min, thus the timeliness of the instrument was not ideal (Wu et al., 2018).

Studies on the analytical techniques based on NIR spectroscopy have played an important role in grain quality detection. However, current studies are still facing the following problems.

1. Commercial instruments mostly consist of spectrometers operating in the full spectral band. They are complex, bulky, expensive to produce, difficult to adapt to different detection requirements, and do not have a grain quality detection model; thus, they are incapable of outputting grain quality information in real-time.
2. Most instruments made in-house consist of spectrometers operating in characteristic spectral bands. The incident light spots of multiple wavelength light sources suffer from the problem of inconsistent detection regions. For grains with uneven distributions of nutritional quality, inconsistent detection regions will result in systematic errors, thereby rendering the instrument unsuitable for grain quality detection.

In this study, a NIR phenotypic sensor about protein and moisture in wheat grain (PSPMWG) was developed. Compared with previous studies, this study made contributions in the following aspects.

1. A NIR PSAGQ was developed, which had a simple structure, was lightweight and was able to detect the grain quality rapidly, nondestructively in real-time. The sensor collects the diffuse reflectance spectrum in real-time and couples the quality detection model to generate grain quality data immediately. In addition, this study expounds on the optical system design and control system development process of the phenotypic sensor in detail, which lays a good foundation for the secondary development of the phenotypic sensor and the application of online detection.
2. A circular structure of multiple light sources was proposed, where the incident light spots of the multiple light sources were consistent, which reduced the measurement error under repeated sample loading conditions caused by the differences in incident light spots. Different incident light spots could be formed by multiple light sources on the detection plane. The overlapping circular part was intercepted to ensure the consistency of incident light spots from multiple light sources and to improve the spectral accuracy of the sensor.
3. A detection model of wheat protein and moisture was built, which was able to output the grain quality information in real-time after obtaining the grain spectrum. The model could be directly used in multiple instruments through the self-correcting luminous state by correcting the reflectance of the sensor through the diffusion fabric and correcting the sample pool. The instrument could be used during grain production, trading, and grain processing.

## Measurement Principles of the Sensor

There are two methods for measuring the NIR: the transmission method (Delwiche et al., 1996; Li et al., 2013) and the reflection method (Hidaka et al., 2011; Priya et al., 2015). The transmission method applies to uniform or transparent samples, and the NIR absorbs the same proportion of light on each equivalent thickness medium on the optical path, that is, the absorbed amount of light is in direct proportion to the number of molecules that generate light absorption on the optical path. The reflection method is mostly used for solid samples, and the NIR passes through a tortuous and irregular optical path in the solid body. The absorbance is related to the scattering coefficient and absorption coefficient of the sample.

Wheat grain is the research object of this study. Its nutritional components are unevenly distributed in the grain. The PSPMWG can obtain the nutritional quality information of the wheat through the grain spectrum measured using the diffuse reflection method.

The relative diffuse reflectance (RDR) is defined as:


(1) $$R=I/I_{0}$$

where I is the diffuse reflection light intensity of the measured sample, and $I_{0}$ is the diffuse reflection light intensity of the background.

Diffuse reflectance spectral signals can also be characterized by absorbance, as defined by


(2) $$A=\log\frac{1}{R}$$

This work used RDR to characterize the diffuse reflectance spectrum. When measuring the diffuse reflectance spectrum, each measurement result of the sample is affected by the loading conditions. Because the internal arrangement and distribution of the samples loaded each time are different, the optical path will change, which will lead to spectral changes and poor spectral repeatability. With the design of a closed sample cell and annular light sources at multiple spectral bands, a NIR PSPMWG could reduce the influence of sample loading conditions on spectral measurement and improve spectral repeatability.

**Figure 1:**
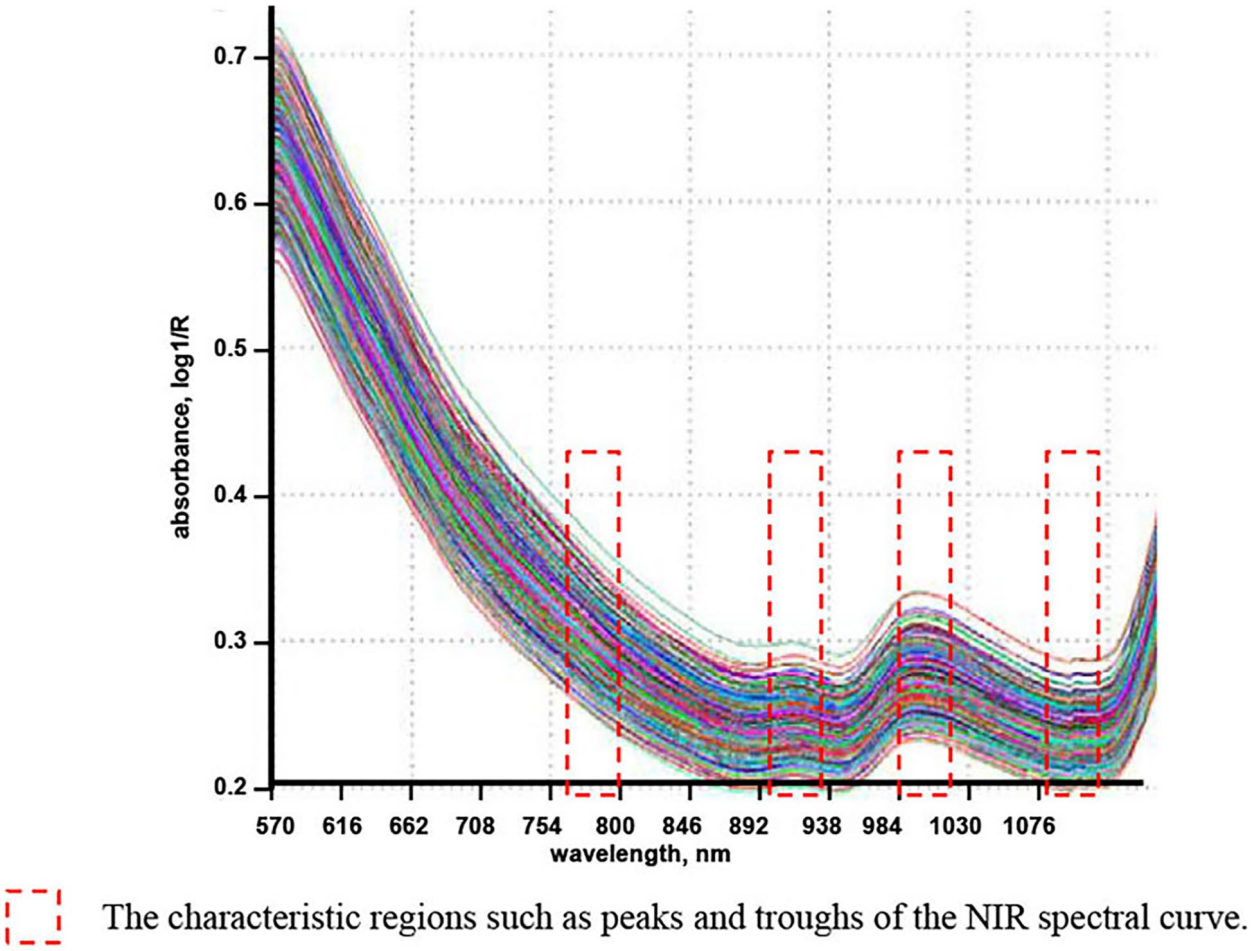
The NIR spectrum of the wheat.

Due to the low manufacturing cost and strong environmental adaptability of spectrometers working in the shortwave infrared and NIR regions (780–1,100 nm; Risius et al., 2014), we used a NIR light source with wavelengths sensitive to grain quality in the shortwave infrared and NIR regions. Figure 1 shows the NIR spectra (570–1,100 nm) of 300 winter wheat samples collected by a Foss InfraXact^™^ Lab/Pro spectrometer (Bec et al., 2020). The regions where the NIR spectral characteristics of the wheat varied were marked by red boxes in the figure. In addition, according to previous studies, (Zhang et al., 2009; Hidaka et al., 2011; Arazuri et al., 2012; Li et al., 2013) on the wavelengths sensitive to grain moisture and protein content, four NIR LEDs with wavelengths of 780, 910, 980, and 1,050 nm were used as the light sources for portable quality detection.

## Design of the Sensor

### Overall Design

**Figure 2:**
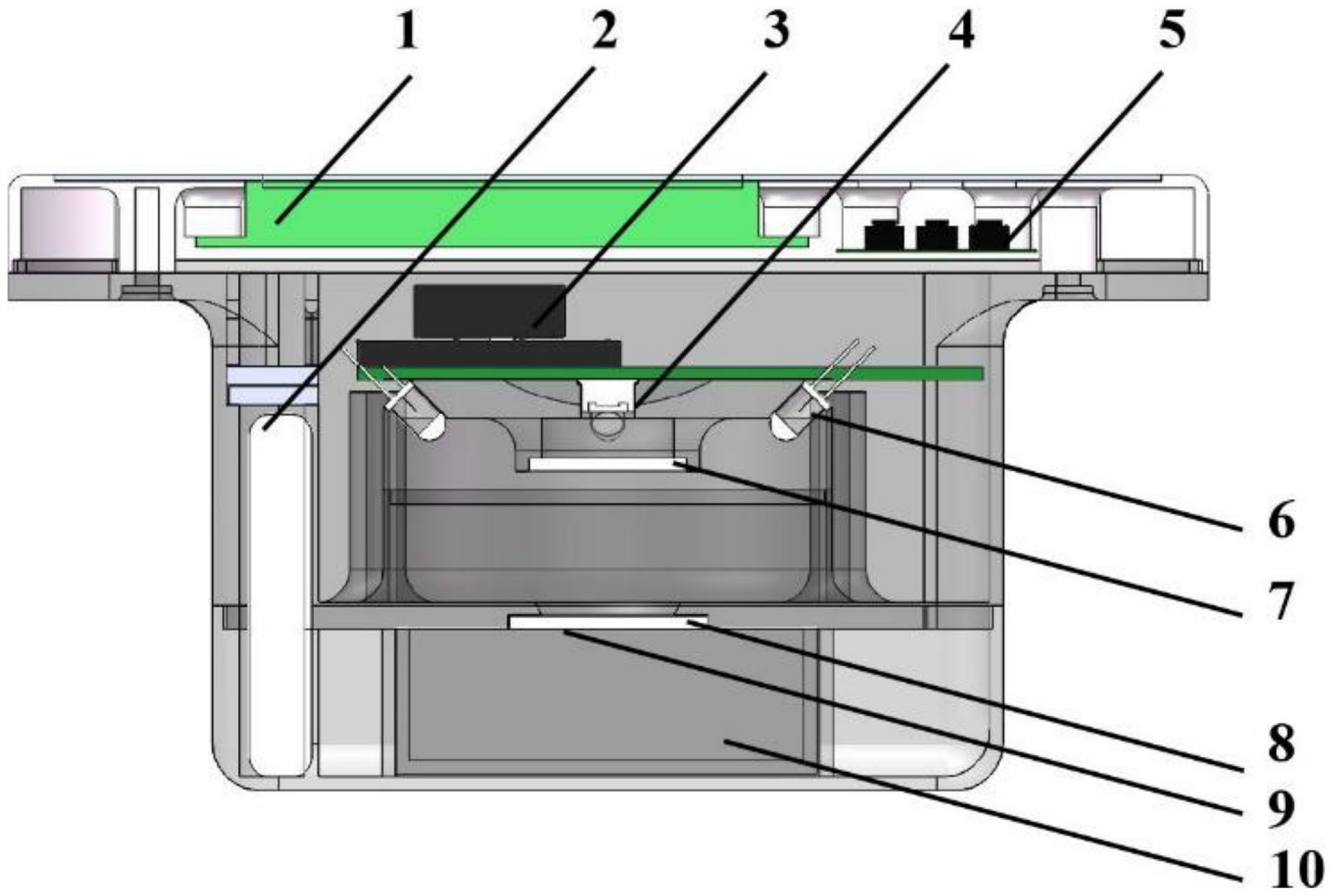
The overall design of the NIR PSPMWG. 1. liquid crystal display (LCD) 2. battery 3. voice broadcast module 4. photoelectric detector 5. button 6. NIR LED 7. Fresnel lens 8. beam shaping diffusion film 9. detection window plane 10. sample pool.

The NIR PSPMWG is composed of an optical system (including a light-emitting unit and a spectral collection unit) and a control system (including hardware circuits and software). The light-emitting unit generates incident spectra, the spectral collection unit obtains the RDR of the grain, and the hardware circuit is used to drive the light-emitting unit and the spectral collection unit. The software couples the RDR with the grain quality detection model to determine the quality content values, which are then displayed on the liquid-crystal display (LCD) with an audible output. The overall structure of the sensor is shown in Figure 2.

### Optical System

#### Overall Design of the Optical System

A stable and reliable optical system is very important for the NIR PSPMWG to obtain the diffuse reflectance spectrum accurately. The light-emitting unit provides a stable and reliable incident spectrum through NIR light sources, and the spectral collection unit collects the diffuse reflectance spectrum of the grain accurately *via* the photoelectric detector.

#### Design of the Light-Emitting Unit

To obtain stable reflectance spectrum signals during grain quality detection, the sensor needs to have light sources with large luminous intensity and stability. Common NIR light sources include a halogen lamp and LED. The halogen lamp is bright and has a long service life, yet it is large and has high power consumption and poor stability of luminous intensity. In addition, the halogen lamp has poor timeliness because it has to be preheated for 30 min before use (Liu et al., 2019). The LED has a long service life, low power consumption, compact dimension, and affordability (Bec et al., 2020). In this study, after comparing the advantages and disadvantages of the two types of light sources, the LED was chosen as the light source for the NIR PSPMWG, with a half-value angle of 12°.

**Figure 3:**
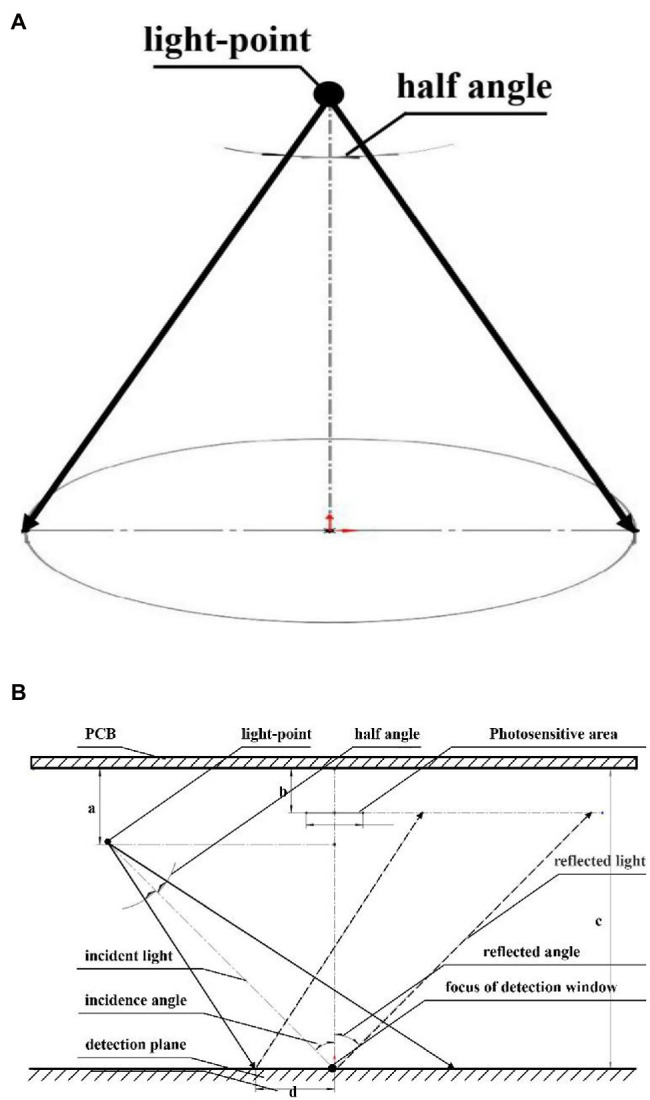
**(A)** Emission light spot of a single LED, **(B)** Emission light spot of LED in the light-emitting unit.

The light-emitting unit was designed based on the selected NIR LEDs, the light-emitting path of the light source is shown in Figure 3A. A circular spot was formed on the vertical plane. The farther away from the light-emitting point, the weaker the light intensity of the spot. Therefore, under a given power consumption, the efficiency of the emission spot could be maximized when the light-emitting point of the light source, the light-sensitive surface center point of the photoelectric detector, and the center point of the detection window plane was on the same plane. In this way, the photoelectric detector could accurately detect the diffuse reflectance spectrum. Moreover, to reduce the influence of the specular reflection light on the collection of reflectance spectrum and to ensure that the specular reflection light is not collected by the photoelectric detector, the incident angle of the LED light was 45°. The design of the light-emitting unit is shown in Figure 3B.

**Figure 4:**
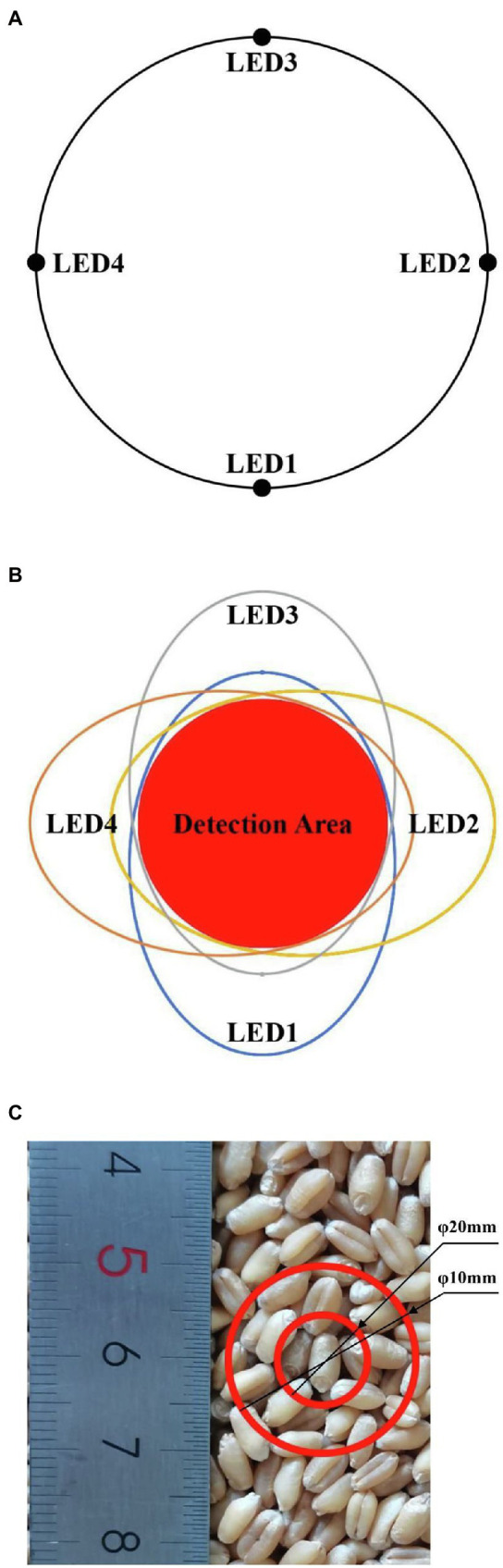
**(A)** The circular layout of four LED light sources, **(B)** Incident light spots and detection window plane, **(C)** Size of the detection window.

**Figure 5:**
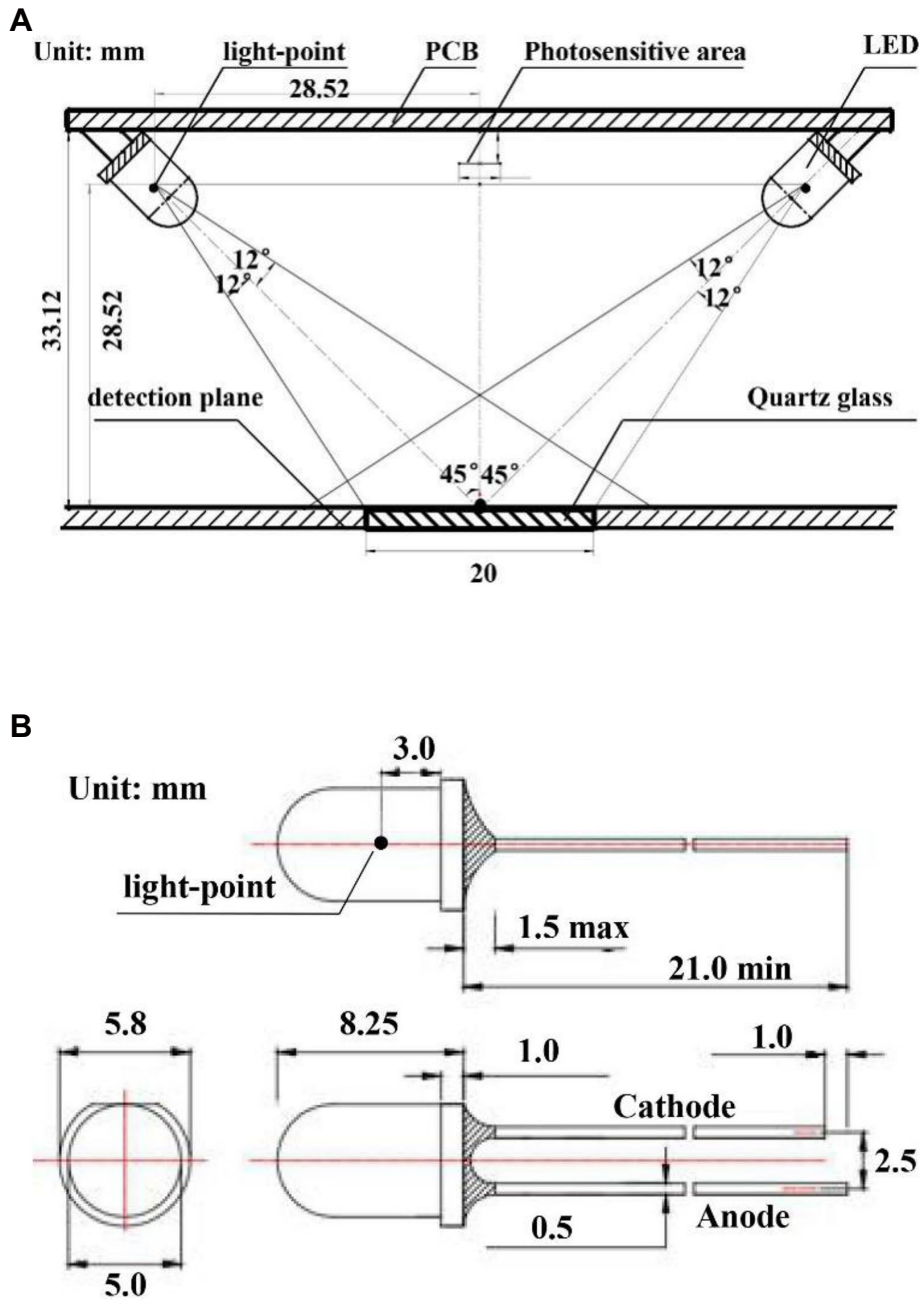
**(A)** Design parameters of the light-emitting unit, **(B)** Outer dimension of LEDs.

According to the above design, the incident light spots of the four light sources are formed in different areas of the detection plane, leading to system errors. Thus, a circumferential layout with even distribution of four LEDs was used (Figure 4A). The incident light spots generated by the four light sources on the detection plane are shown in Figure 4B. The incident light spot areas formed by the four LEDs were different, yet there were some overlapping areas. The overlapping areas of the four incident light spots were chosen as the detection window to ensure consistency in the detection area of the four light sources and to reduce system error. The nutritional components of wheat were distributed unevenly, thus there might be random errors in the detection of single-grain wheat. There should be multiple wheat grains in the detection area so that the diffuse reflectance spectrum can effectively lower random errors. The dimensions of the detection window are shown in Figure 4C. When the detection window is a circle with a diameter of 10 mm and there was only one single grain of wheat, there might be a random error. When the detection window was a circle with a diameter of 20 mm and there were 10 grains in the window, the random error seen in single-grain wheat detection was lowered effectively. Therefore, the detection window was set to a circle with a diameter of 20 mm, and the optical path and grain were separated by quartz glass. The specific design parameters of the light-emitting unit are shown in Figure 5.

**Figure 6:**
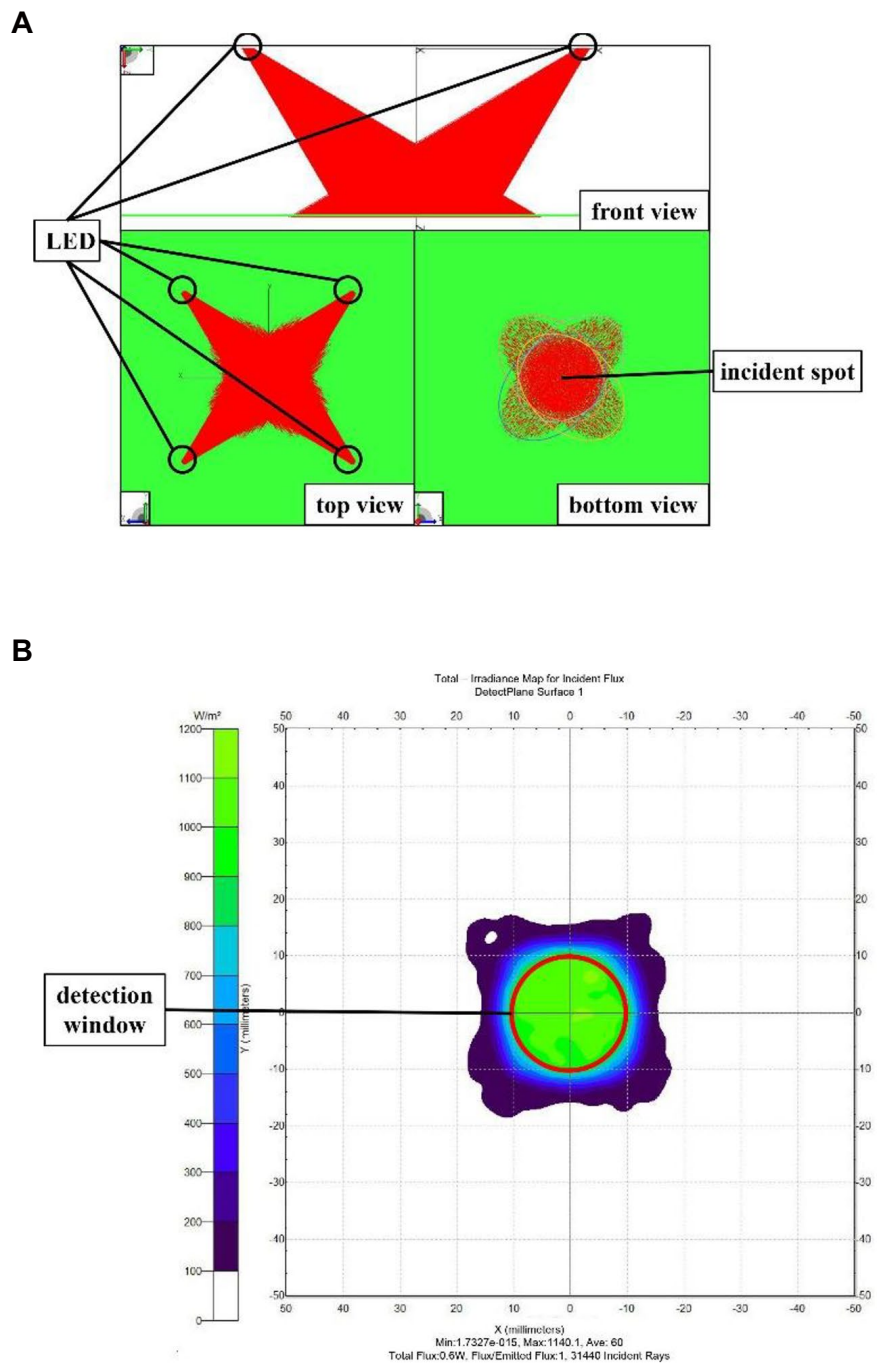
**(A)** TracePro simulation of the light-emitting unit, **(B)** Irradiance analysis of the detection plane.

The light-emitting unit was simulated and analyzed by the TracePro optical simulation software to verify the consistency of the incident light spots by the four light sources. First, four light sources with an incident angle of 45° and a half-value angle of 12° were constructed, and a light beam blocking plate was placed at the appropriate location to substitute the detection plane in the actual design. The simulation results of the light-emitting system are shown in Figure 6A, where the bottom view showed that the elliptical light spots of the four light sources formed a circular light coincidence area on the detection surface (the same sample area detected with the four light sources). This circular area was used as the detection window to ensure the consistency of the incident light spots from the four light sources. The irradiance analysis diagram from the light beam blocking plate (detection plane) is shown in Figure 6B, where the location of the detection window is marked by a red circle in the figure.

#### Design of the Spectral Collection Unit

A photoelectric detector is an electronic component that converts optical signals to electric signals (Yang et al., 2005), which ensures the accurate collection of the diffuse reflectance spectrum. The selection of a NIR photoelectric detector is mainly determined by three factors: response scope, response speed, and sensitivity. In this study, the spectral response of the silicon photoelectric detector ranged from 320 to 1,100 nm, the response time was 3.6 μs, and the sensitivity for the four wavelengths was above 0.4 A/W. These parameters satisfied the requirement of the NIR PSPMWG. The light emitted from the four LEDs could be converted into current by the photodiode quickly and accurately.

**Figure 7:**
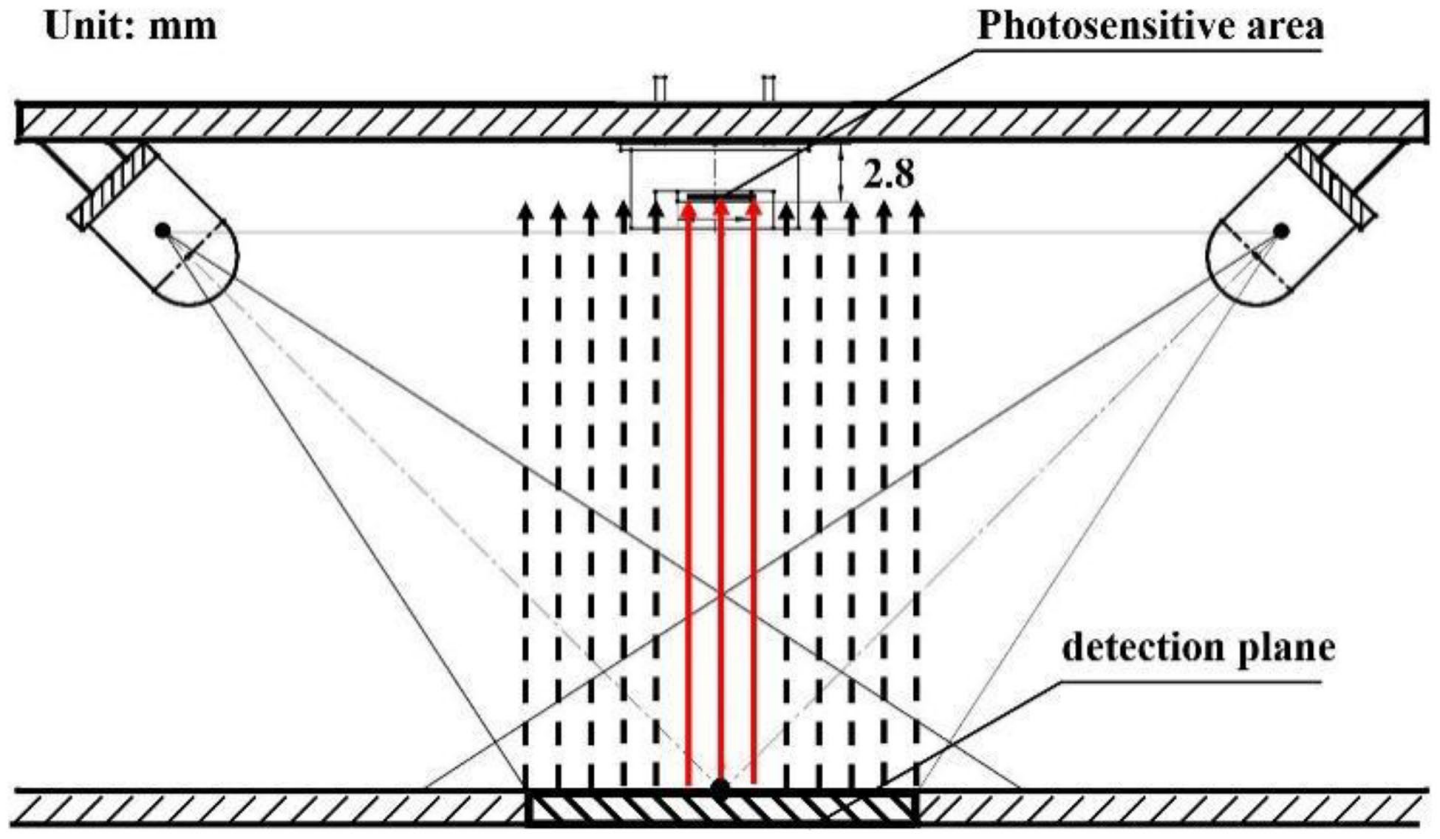
Direct spectrum acquisition of diffuse reflection tube.

The spectral collection unit was designed based on the selected photoelectric detector. The diffuse reflectance spectrum emitted from the detection window was a circle with a diameter of 20 mm, while the photosensitive area of the selected photoelectric detector was a 3.6 × 3.6 mm^2^. The collection of diffuse reflectance spectrum by the photoelectric detector is shown in Figure 7. The photoelectric detector only collects the diffuse reflectance spectrum that is received by the photosensitive area; it does not collect the other diffuse reflectance spectrum, leading to low accuracy in the diffuse reflectance spectrum.

**Figure 8:**
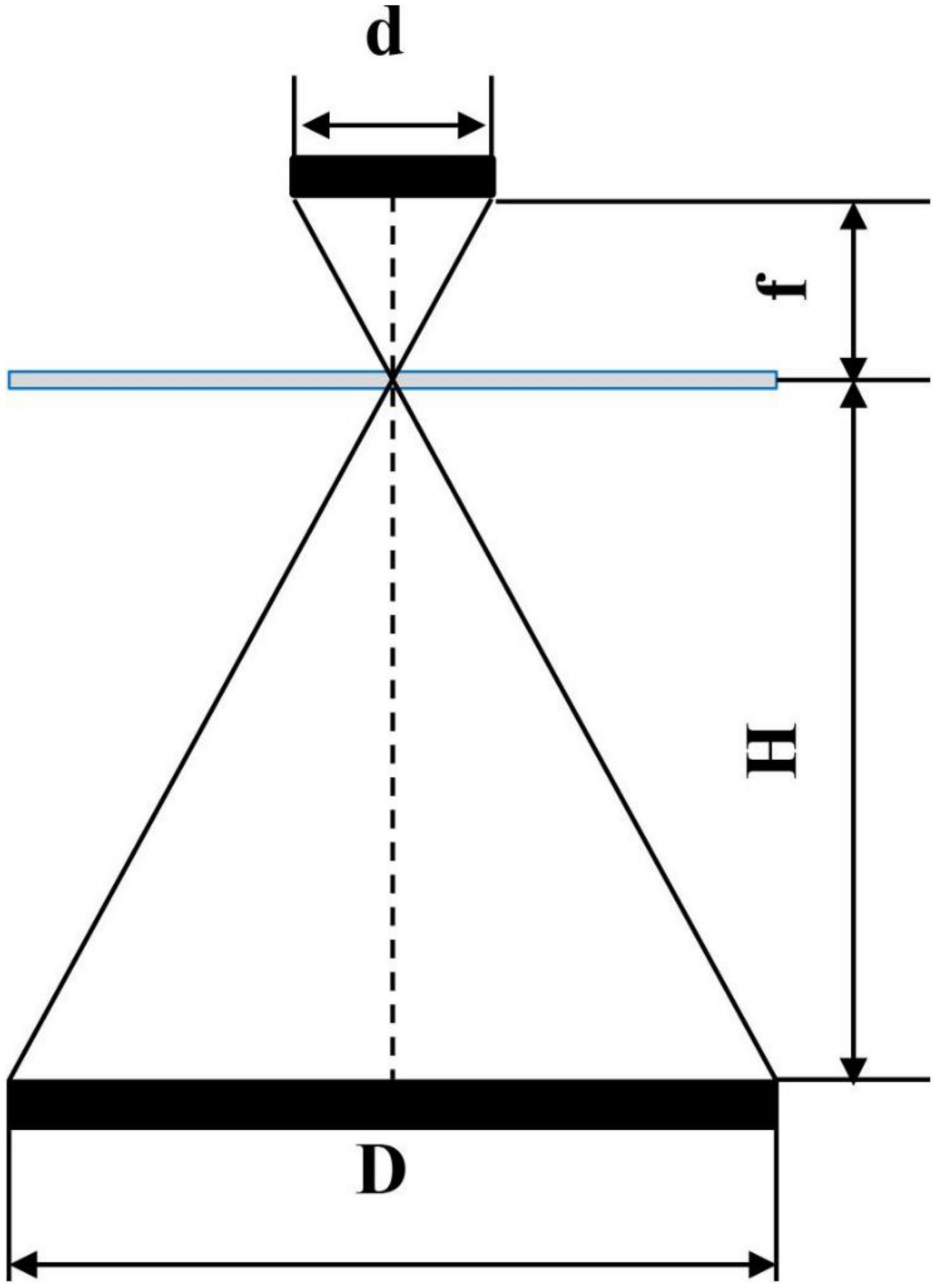
Inductive light.

To improve the collection accuracy in the diffuse reflectance spectrum, all diffuse reflectance spectrum of grain has to be received by the photoelectric detector, that is, the circular diffuse reflectance spectrum with a diameter of 20 mm needs to be reflected on the square photosensitive area. Therefore, it is necessary to add a condenser between the detection window and the photoelectric detector. The Fresnel lens is developed based on the Fresnel theory (Wu et al., 2018), it is small and light and is suitable for the application of the sensor. The diffuse reflectance spectrum is condensed as much as possible without blocking the incident light, and the Fresnel lens has the same dimensions as the detection window, i.e., a circle with a diameter of 20 mm. The schematic diagram of the photosensitive optical path is shown in Figure 8.

The calculation of the focal length of the lens is as follows:


(3) $$\frac{f}{d}=\frac{H}{D}$$

where D is the dimension of the reflection spot, d is the dimension of the photosensitive element, and H is the distance from the light spot to the lens.

**Figure 9:**
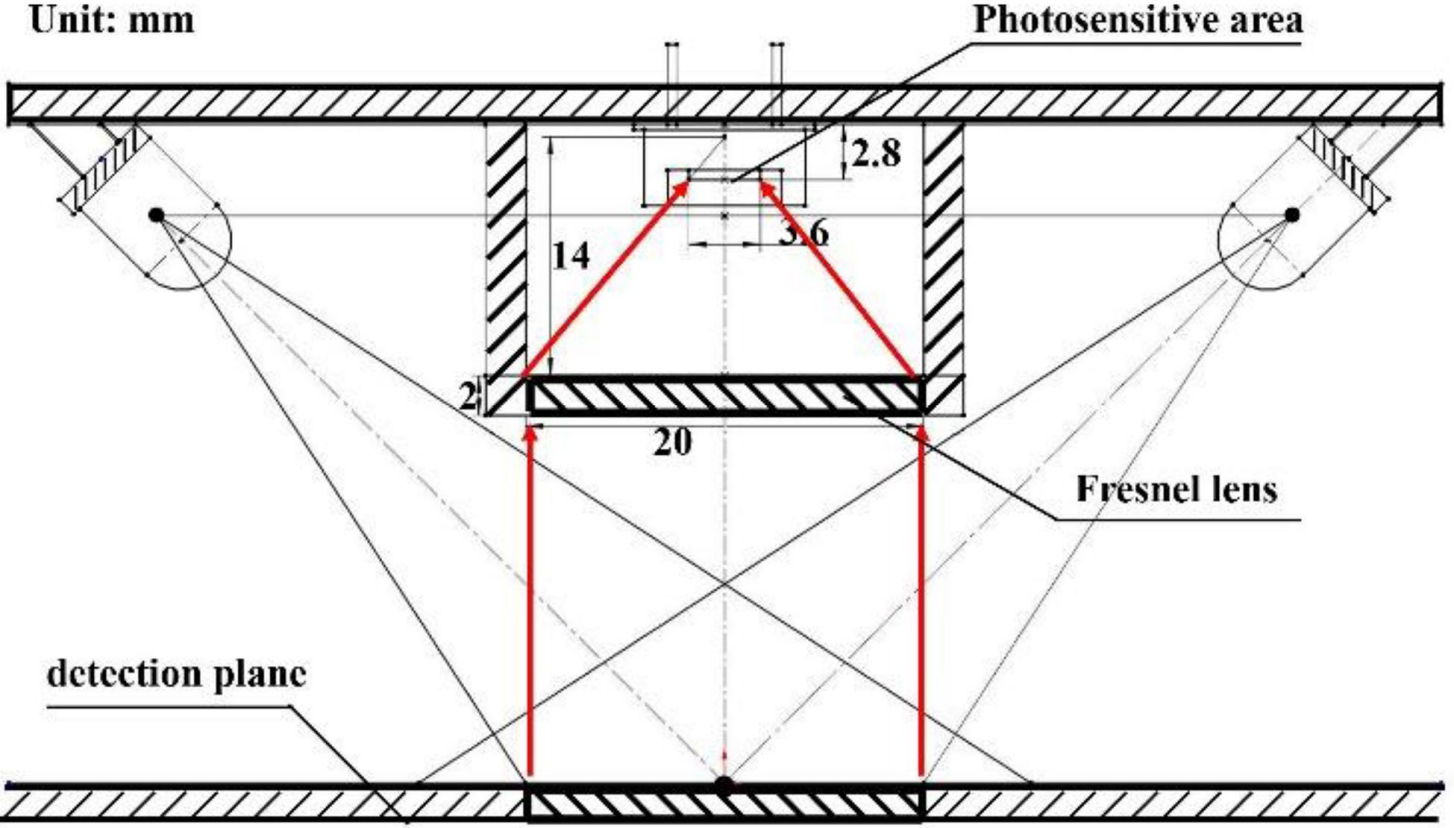
Spectral acquisition unit design.

Based on the dimensions of the spectrum collection unit, the focal length of the Fresnel lens was 15 mm, and the diameter was 20 mm, with a thickness of 2 mm. The above parameters were substituted to the calculation formula (Yao et al., 2020) of Fresnel lens to calculate the intersection angle between the lens sawtooth and the vertical direction, which was 2.67°. The spectral collection unit is shown in Figure 9.

**Figure 10:**
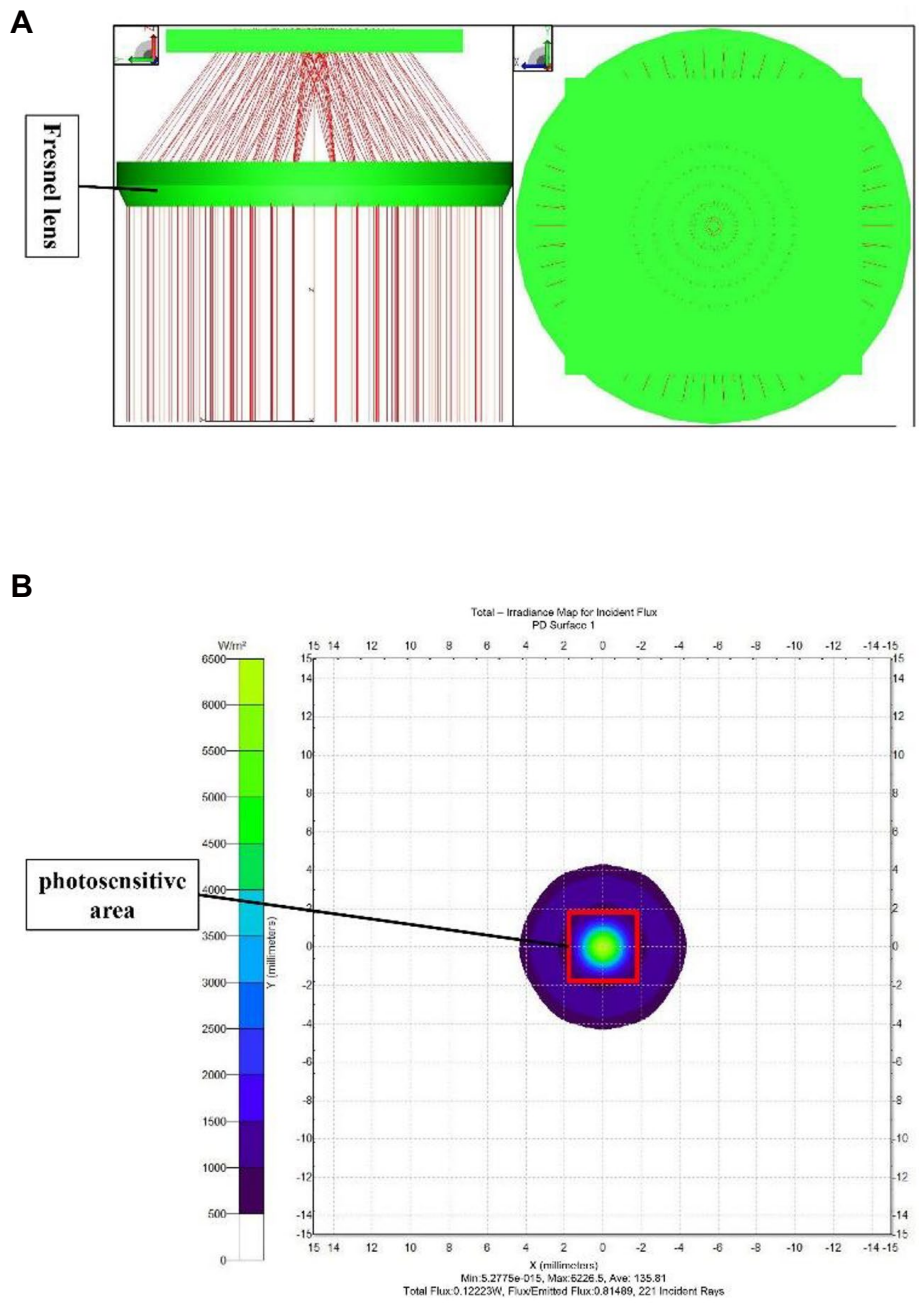
**(A)** TracePro simulation of the spectrum acquisition unit, **(B)** Irradiance analysis diagram of the photosensitive plane.

The spectrum acquisition unit was simulated and analyzed by the TracePro optical simulation software to verify the light-condensing performance of the Fresnel lens used in the system. The Fresnel lens was constructed based on a theoretical design, and a light-blocking plate was placed at an appropriate location to replace the photosensitive plane in the actual design. Then, the light-condensing performance of the lens was assessed by a parallel light beam source. The simulation results of the spectrum acquisition unit are shown in Figure 10A. The parallel light beam was converged by the lens onto the light-blocking plate (photosensitive plane), with most of the light falling within an area of 3.6 × 3.6 mm2. The photodetector placed in this area could accurately collect the convergent diffuse reflection spectrum. The irradiance analysis diagram of the light-blocking plate (photosensitive plane) is shown in Figure 10B, where the position of the photosensitive plane of the photosensitive element is marked by a red box in the figure.

### Control System

#### Overall Design of the Control System

The control system consisted of hardware circuitry and a software system. The hardware circuit is composed of the light source driver circuit, the spectral collection circuit, and the signal conditioning circuit. The hardware circuit is mainly used to ensure stable luminescence of the light source and accurate collection of the diffuse reflectance spectrum of grain. The software system mainly achieves the collection of the diffuse reflectance spectrum of grain, couples that data with the grain quality prediction model, and outputs the grain quality results.

#### Hardware Circuit Design

**Figure 11:**
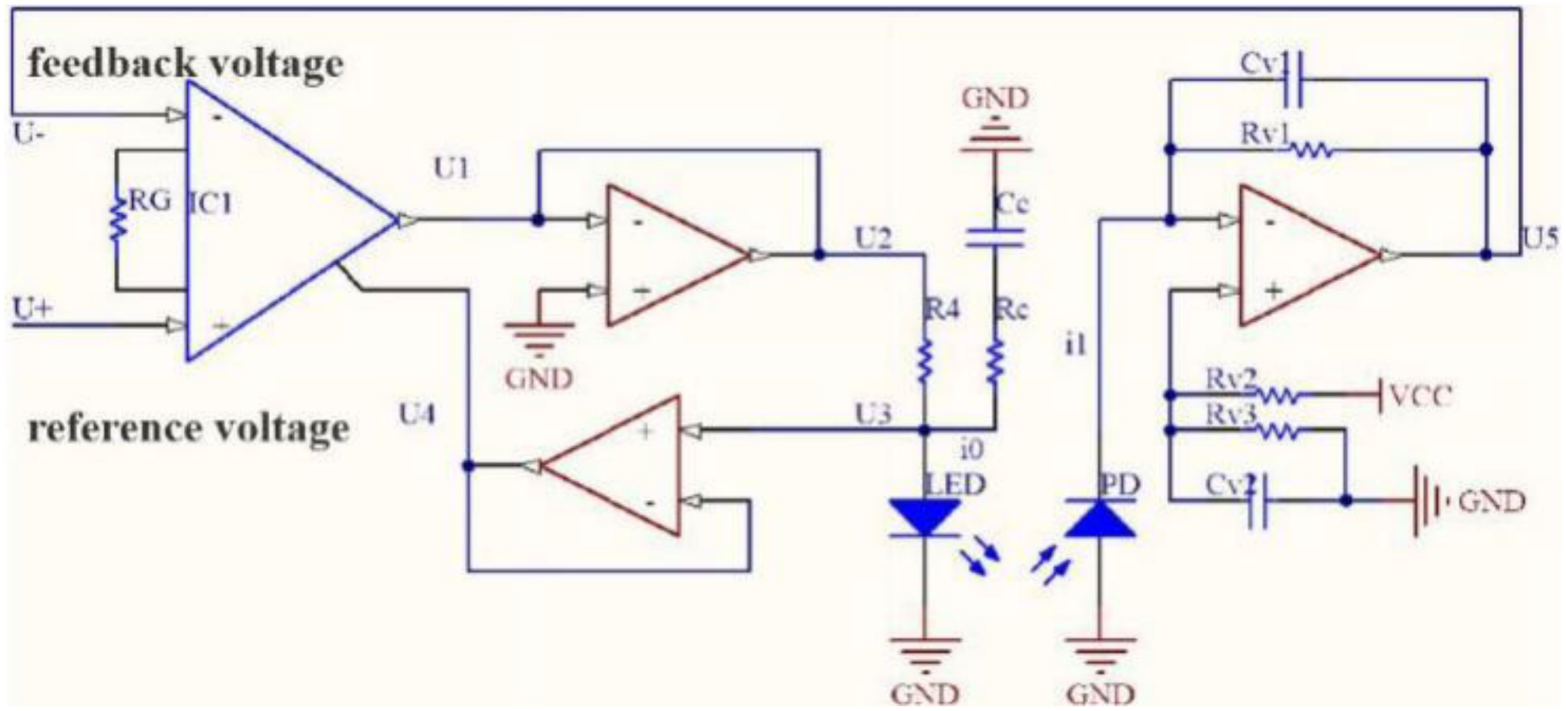
Optical power feedback drive circuit.

The luminous intensity of the light-emitting unit needs to be stable for the PSPMWG to accurately collect the diffuse reflectance spectrum. The luminous intensity of the light-emitting unit is affected by its factors (e.g., light source loss and heat dissipation) and driver factors such as drive current. The light source is driven by the optical power feedback circuit. The light source driver circuit based on optical power feedback mainly consists of four parts: optical power feedback, comparative amplification, constant current drive, and compensation filter. The circuit diagram is shown in Figure 11.

When the luminous power of the light source under constant current reduces, the feedback voltage in the optical power feedback circuit decreases, the drive voltage in the comparative amplification part increases, the drive current in the constant current driving part increases, and the luminous power of the driving light source increases, and vice versa. In the compensation filter part, the light source drive signal is stabilized by reducing the ringing and overshoot driven by the modulation signal through the resistive–capacitive compensation filter circuit. The drive current and luminous power of the light source can be balanced through the optical power feedback to achieve stable luminous power of the light source.

The accuracy of the grain diffuse reflectance spectrum obtained by the spectrum collection circuit directly affects the accuracy of grain quality data. In the spectrum collection circuit, the diffuse reflectance spectrum is first converted into light current through the photoelectric detector, which is converted into a voltage signal using the current–voltage conversion circuit, then the noise signal is filtered. Lastly, the voltage signal is amplified to the collection range of the microprocessor for further processing and output.

**Figure 12:**
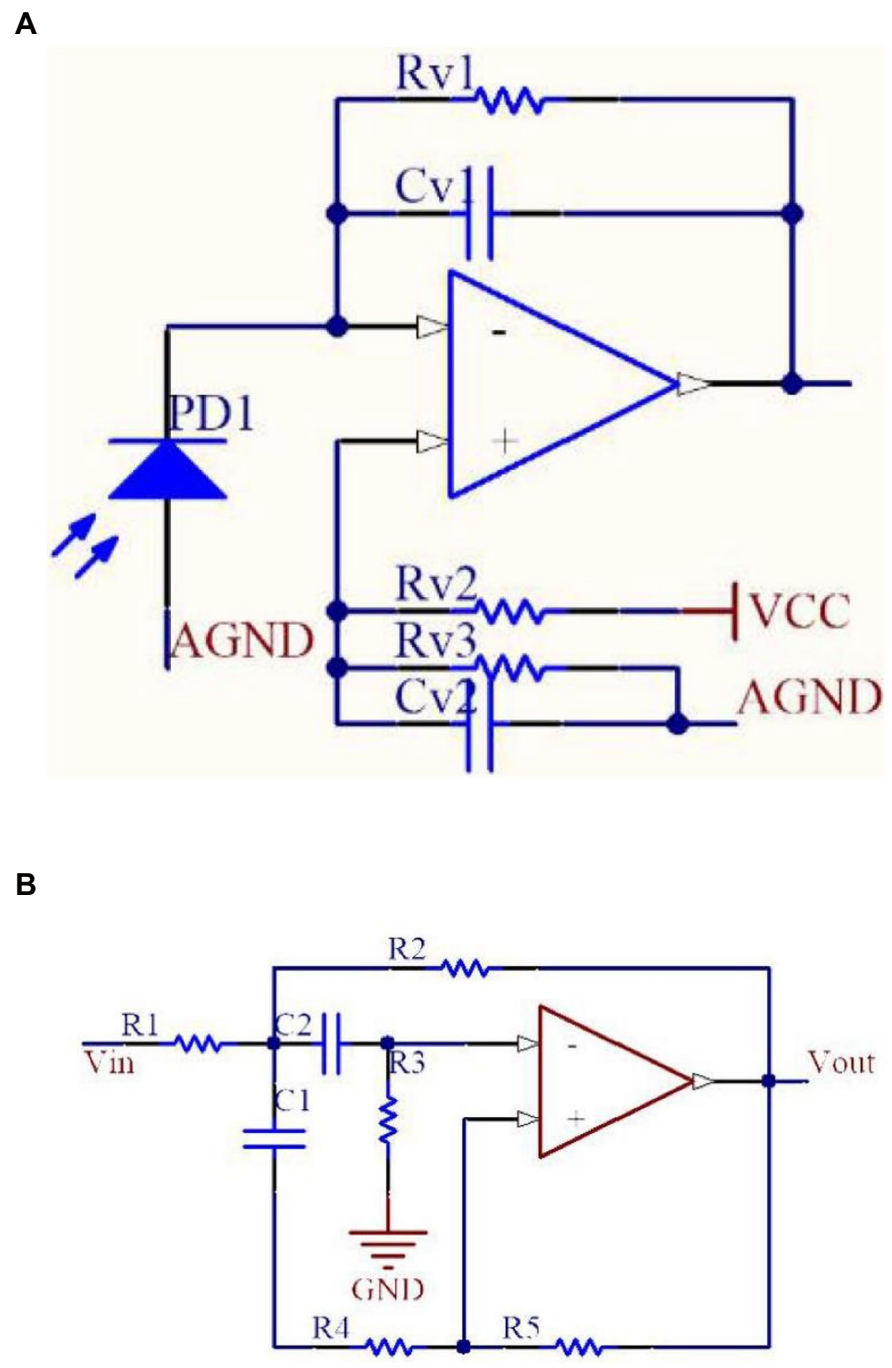
**(A)** Current–voltage conversion circuit, **(B)** MFB-type second-order band-pass filter circuit.

The spectrum collection circuit mainly consists of three parts: current–voltage conversion, filtering, and amplification. The current–voltage conversion circuit is shown in Figure 12A. The resistance, capacitance, and integrated operational amplifier constitute the photodiode transimpedance amplifier. The resistances RV2 and RV3 and capacitance CV2 constitute the bias voltage with a positive power supply, which is connected to the positive phase input end of the integrated operational amplifier to avoid circuit instability resulting from the saturation of the negative power supply rail when there is no current. The filter circuit is shown in Figure 12B. The active filter circuit online design tool Filter Design Tool of the Texas Instruments Company was used to implement circuit design (Duff and Kalb, 2015).

#### Software System Design

**Figure 13:**
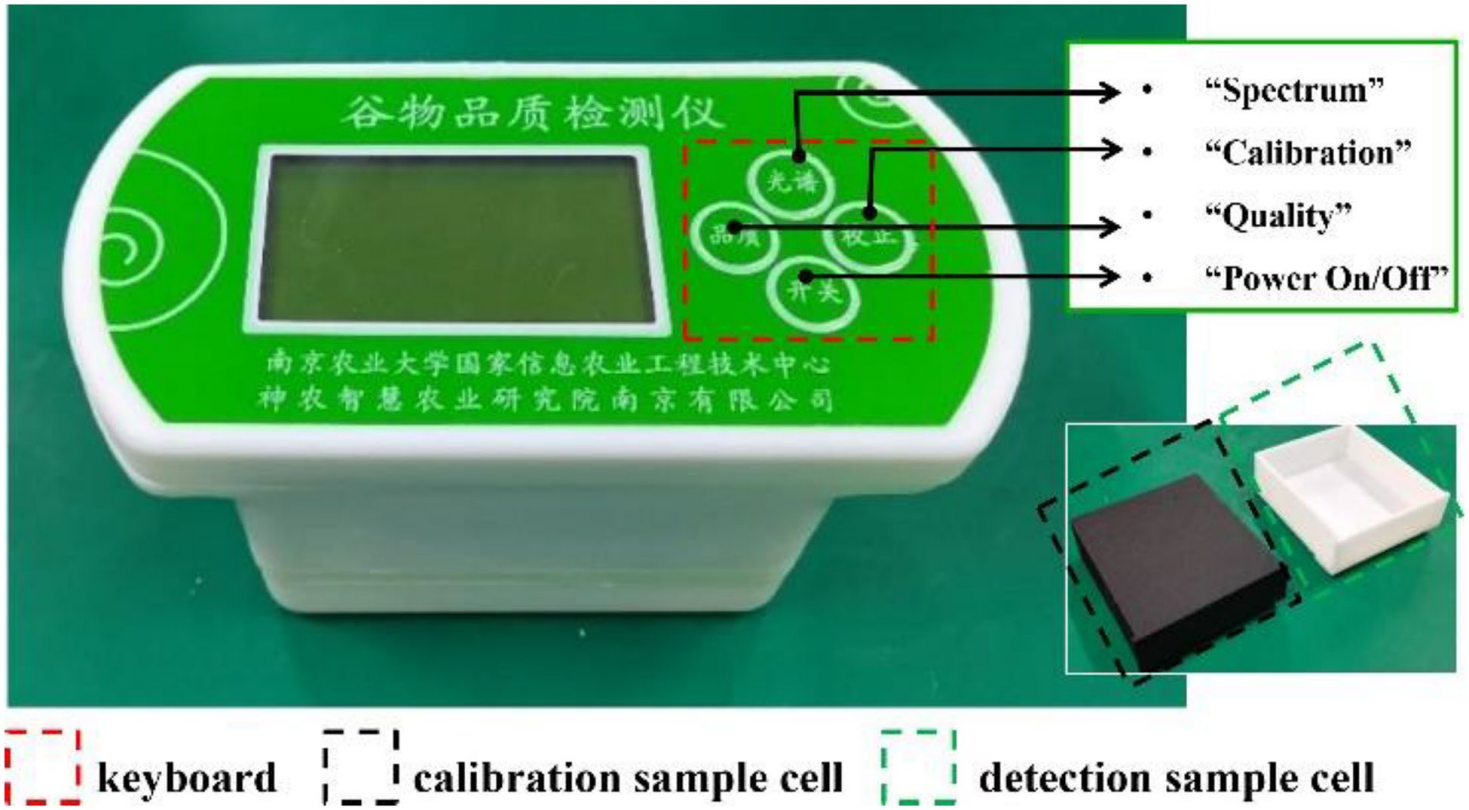
The physical image of the NIR PSPMWG.

The software system is composed of two modules: initialization and functional control. The initialization module is used for microprocessor initialization, pulse width modulation (PWM) signal initialization, digital-to-analog conversion initialization, keyboard input, and LCD initialization. The functional control module is used for light source time-sharing drive, diffuse reflectance spectrum collection, diffuse reflectance spectrum correction, grain quality detection, keyboard input control, LCD output, and audible output. Light source time-sharing driving means that the digital input/output ports of the microprocessor generate PWM signal and light source control signals, and then the PWM signals make the LEDs generate the modulated light signals. Diffuse reflectance spectrum collection is the collection of modulated light signals through a spectrum collection circuit. Diffuse reflectance spectrum correction is performed by correcting the luminous state of the LEDs in real-time through the correction sample cell (Figure 13). Grain quality detection is the coupling of diffuse reflectance spectrum and grain quality detection model to calculate quality components in real-time. And, the grain quality detection model is converted into the quality detection library file in c format through MATLAB coder, and the quality detection library file is called in the microprocessor program to realize real-time detection.

As shown in Figure 13, the NIR PSPMWG has three functions: “correction,” “spectrum” and “quality.” In the “correction” mode, the function of correcting the diffuse reflectance spectrum is realized. In the “spectrum” mode, the light source is first driven by time-sharing, and then the diffuse reflectance spectrum is acquired in real-time through the diffuse reflectance spectrum collection; In the “quality” mode, the grain quality information is generated in real-time through the grain quality detection model, and then displayed through the LCD and output through speech synthesis.

## Tests and Results

### Tests

Three types of tests were conducted: correction, performance, and calibration. In the correction test, multiple PSPMWGs were used to measure the relationship between the RDR and the standard diffuse reflectance of the diffusion fabric. The purpose is to ensure the universal application of the grain quality detection model in multiple PSPMWGs. Diffusion fabrics with standard diffuse reflectance of 6.5, 25, and 48% were used as the test material in the correction test. The RDR of the diffusion fabrics was measured by the PSPMWG, and the mean value of each diffusion fabric measured five times was taken as its RDR. Then, the linear relationship between the RDR and the standard diffuse reflectance of the diffusion fabric was established.

In the performance test, the RDR of the same grain was measured several times to verify the stability of the NIR PSPMWG. Two wheat grain samples (sample 1: Yangmai 23, sample 2: Ningmai 13) were selected in the performance test. The PSPMWG was used to collect the noise level of the RDR instrument five times, and the repeatability performance of the RDR instrument was accessed under the repeated sample condition.

In the calibration test, a relational model between the RDR and the physicochemical analyses values was built such that the moisture and protein contents could be directly obtained once the RDR was measured by the PSPMWG. Twenty-four types of wheat grain samples (a total of 48 samples, each variety had two grains, and each sample weighed 250 g) were selected in the calibration test. The PSPMWG was used to collect the RDR of wheat grain; the mean value of each sample measured five times was taken as the RDR, and the moisture and protein contents of 48 samples were measured *via* physicochemical analyses. Then, the relational model between RDR and physicochemical analyses values was created using the chemometrics method, i.e., the wheat grain quality detection model.

### Materials

**Figure 14:**
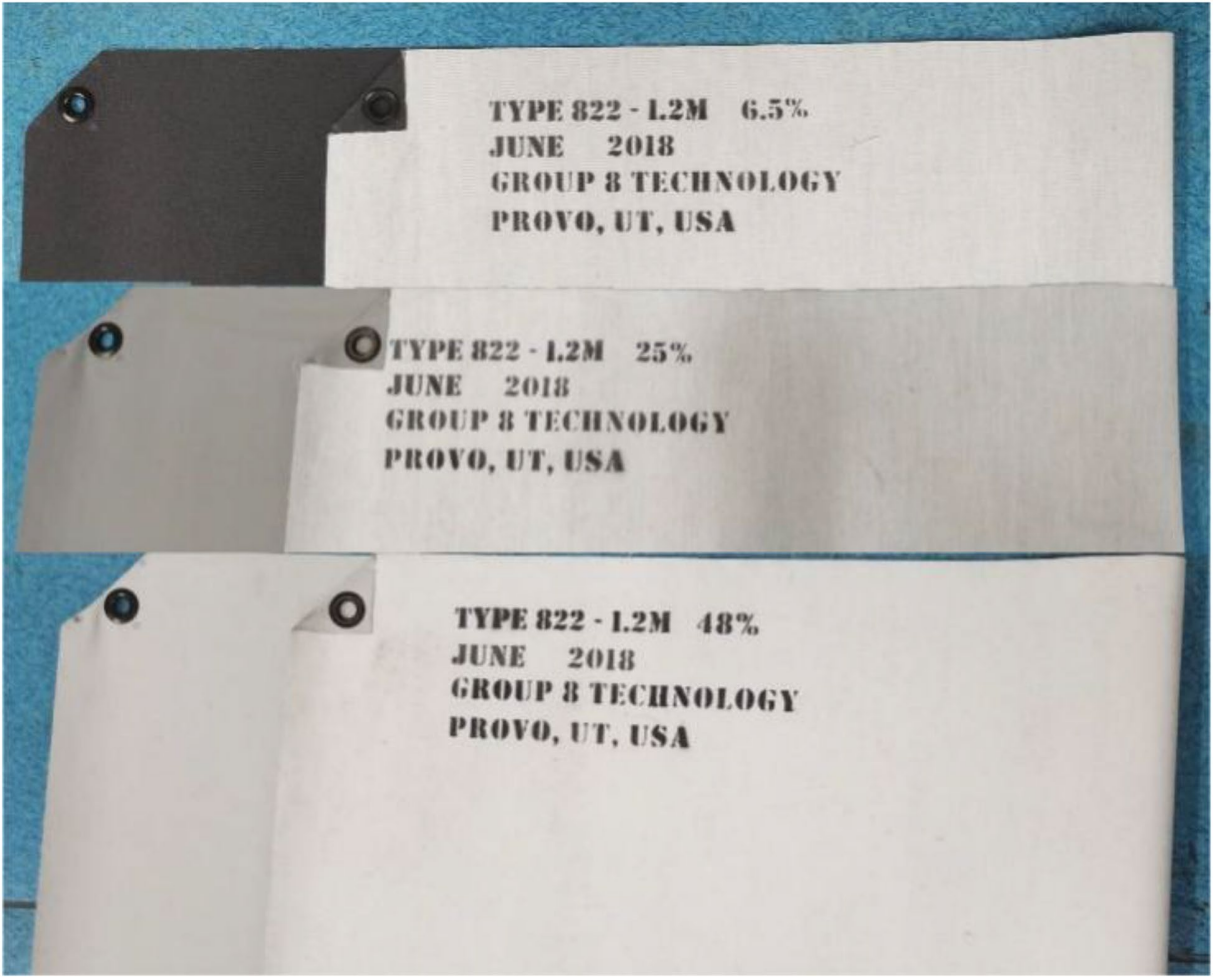
Three diffusion fabrics.

The test equipment includes PSPMWG and diffusion fabrics. A physical image of the NIR PSPMWG is shown in Figure 13. The phenotypic sensor can obtain the RDR of four wavelengths in real-time, i.e., 780, 910, 980, and 1,050 nm. The diffuse reflectance of the three diffusion fabrics was 6.5, 25, and 48%. A physical image is shown in Figure 14.

### Determination of Quality Parameters

In the physicochemical analyses of moisture and protein contents of wheat, the conventional ISO method (ISO, 2010) was used to determine the moisture of wheat. Specifically, the sample was weighed on a dried aluminum container with constant weight and dried at (130 ± 3) °C for 90 min. Then, the sample was taken out and cooled to air temperature in the dryer, which was weighed again. The protein content of wheat was determined by the semi-micro Kjeldahl method (ISO International Standard, 2006), and the protein content was calculated by multiplying the nitrogen content by 5.7. The moisture and protein contents of each sample were measured twice, and the mean value was taken as the final data.

### Modeling Methods

**Figure 15:**
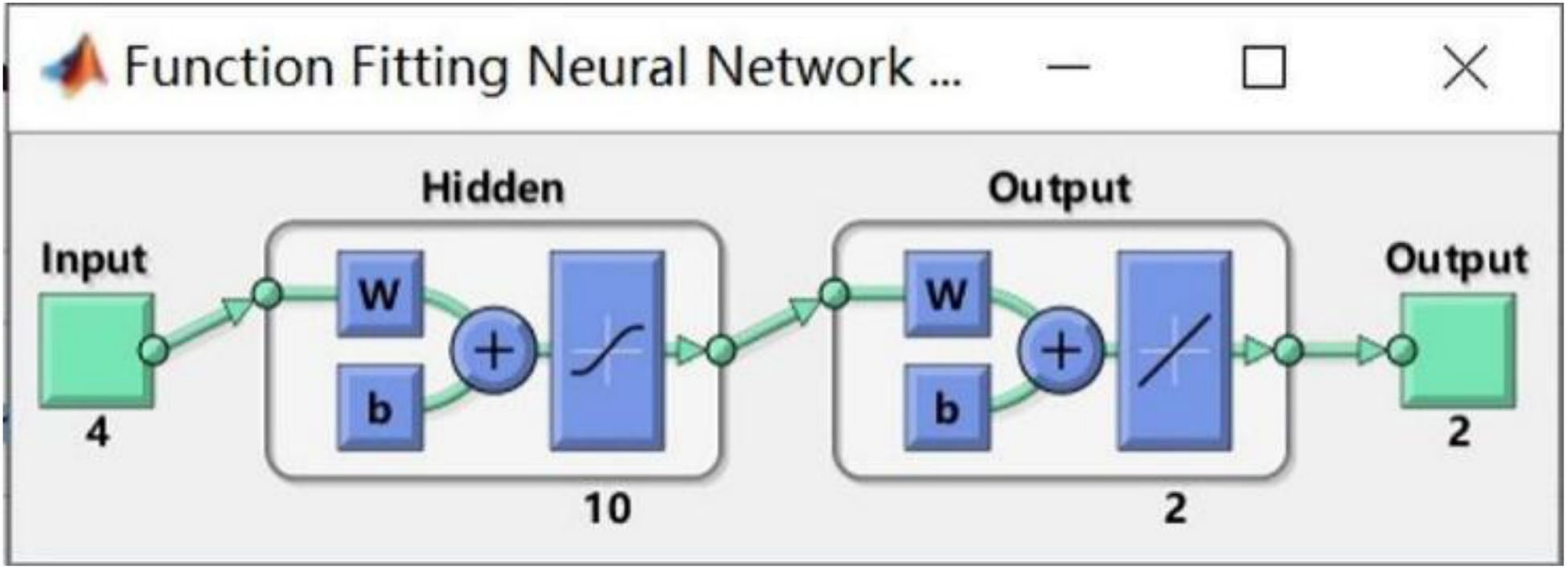
Neural network diagram.

There was a nonlinear relationship between the RDR of wheat grain and its physicochemical analyses values. Due to the interaction between the moisture of wheat grain and the components such as protein, the noise of the sensor and other factors can also cause nonlinearity, and the ideal model cannot be obtained by the linear correction method. The artificial neural network method has high nonlinear expression ability and is widely used to establish nonlinear NIR analysis models. In the PSPMWG, the Neural Fitting (nftool) app in MATLAB was used to establish the grain quality, detection model. The nftool is a two-layer feed-forward network with sigmoid hidden neurons and linear output neurons, that can fit multi-dimensional mapping problems arbitrarily well, neural network is shown in Figure 15. 48 samples were randomly divided into a Training set (28 samples), Validation set (10 samples), and Test set (10 samples). In the nftool, the Training set is presented to the network during training, and the network is adjusted according to its error; the Validation set is used to measure network generalization, and to halt training when generalization stops improving, and the Test set provides an independent measure of network performance during and after training. The RDR of wheat grain was used as the input, and the moisture and protein content were used as the output. The model was evaluated using the coefficient of determination (*R*^2^), RMSE, and MAE.

(1) (R2)


(4) $$R^{2}=1-\frac{\sum_{i=1}^{n}{\left(y_{i\_true}-y_{i\_predict}\right)}^{2}}{\sum_{i=1}^{n}{\left(y_{i\_true}-{\bar{y}}_{true}\right)}^{2}}$$

where $y_{i\_\mathrm{true}}$ is the physicochemical value of sample *i*, $y_{i\_\mathrm{predict}}$ is the predicted value of sample i, ${\bar{y}}_{\mathrm{true}}$ is the average physicochemical value of all samples, and *n* is the number of samples. With the same real value range, the closer R^2^ is to 1, the better the regression or prediction of the model.

(2) RMSE


(5) $$\mathrm{RMSE}=\sqrt{\frac{\sum_{i=1}^{n}\left(y_{i\_\mathrm{true}}-y_{i\_\mathrm{predict}}\right)2}{n}}$$

where $y_{i\_\mathrm{true}}$ is the physicochemical value of sample i, $y_{i\_\mathrm{predict}}$ is the prediction value of sample i, and n is the number of samples. The smaller RMSE, the better predictability of the model (Hussain et al., 2019).

(3) MAE


(6) $$\mathrm{MAE}=\frac{\sum_{i=1}^{n}\left|y_{i\_\mathrm{true}}-y_{i\_\mathrm{predict}}\right|}{n}$$

where $y_{i\_\mathrm{true}}$ is the physicochemical value of sample i, $y_{i\_\mathrm{predict}}$ is the prediction value of sample i, and n is the number of samples. The smaller MAE, the better predictability of the model. MAE can reflect the actual situation of the predicted value error. The smaller MAE, the smaller the prediction error.

### Results and Discussion

#### Correction Test

**Figure 16:**
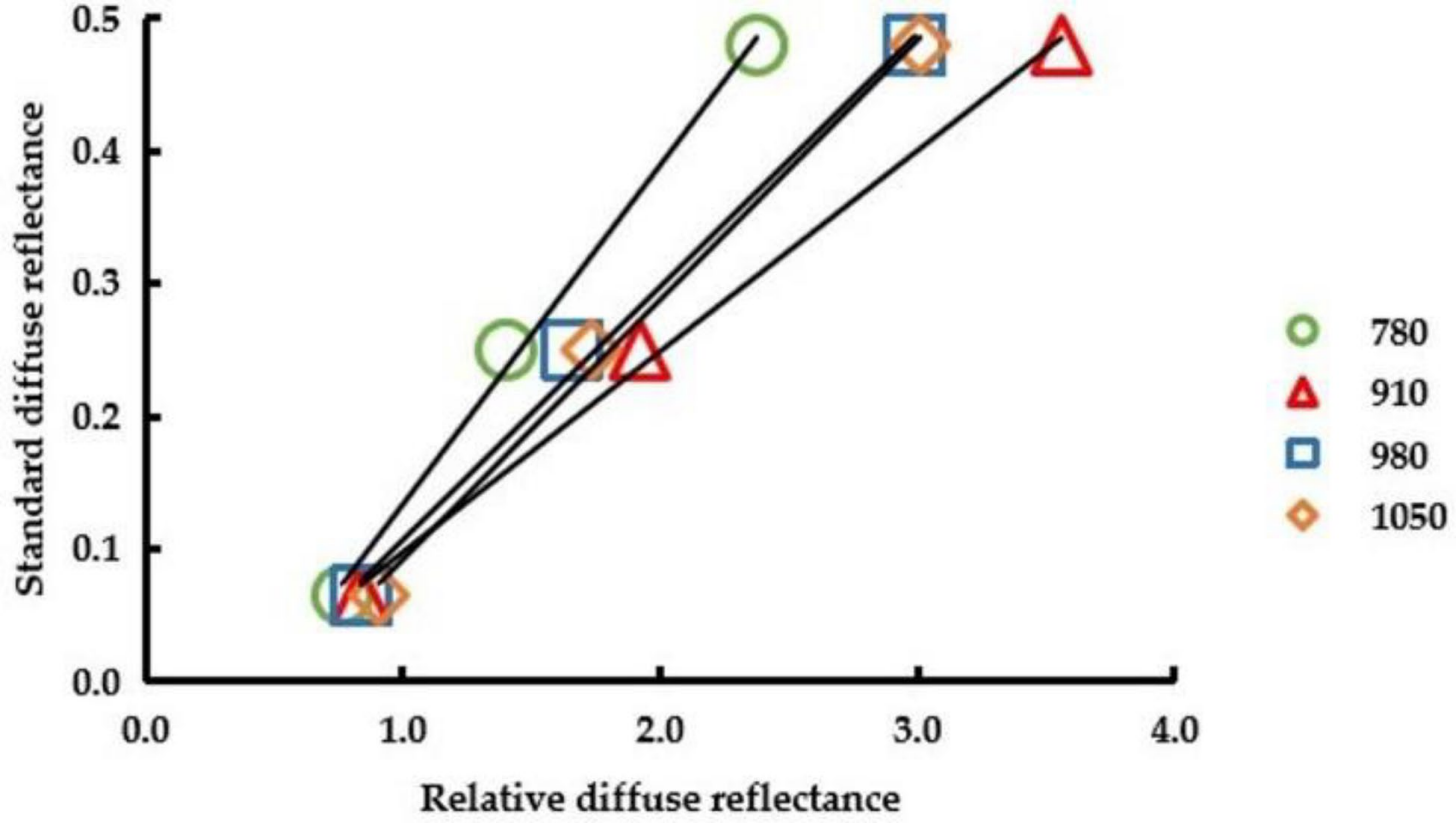
Diffuse reflectance correction of the PSPMWG.

The diffuse reflectance correction of the NIR PSPMWG is shown in Figure 16. Here, 780, 910, 980, and 1,050 refer to the diffuse reflectance corresponding to the wavelengths of 780, 910, 980, and 1,050 nm, respectively. The RDR of the diffuse fabrics collected by the phenotypic sensor was used as the independent variable and the standard reflectance of the diffuse fabrics was used as the dependent variable; then, the diffuse reflectance correction equation was established. The values of *R*^2^ were all above 0.99. The diffuse reflectance correction equations of 780, 910, 980, and 1,050 nm were *y* = 0.2560 × −0.1227, *y* = 0.1515 × −0.0541, *y* = 0.1911 × −0.0845, and *y* = 0.196 × −0.1042.

#### Performance Test

**Figure 17:**
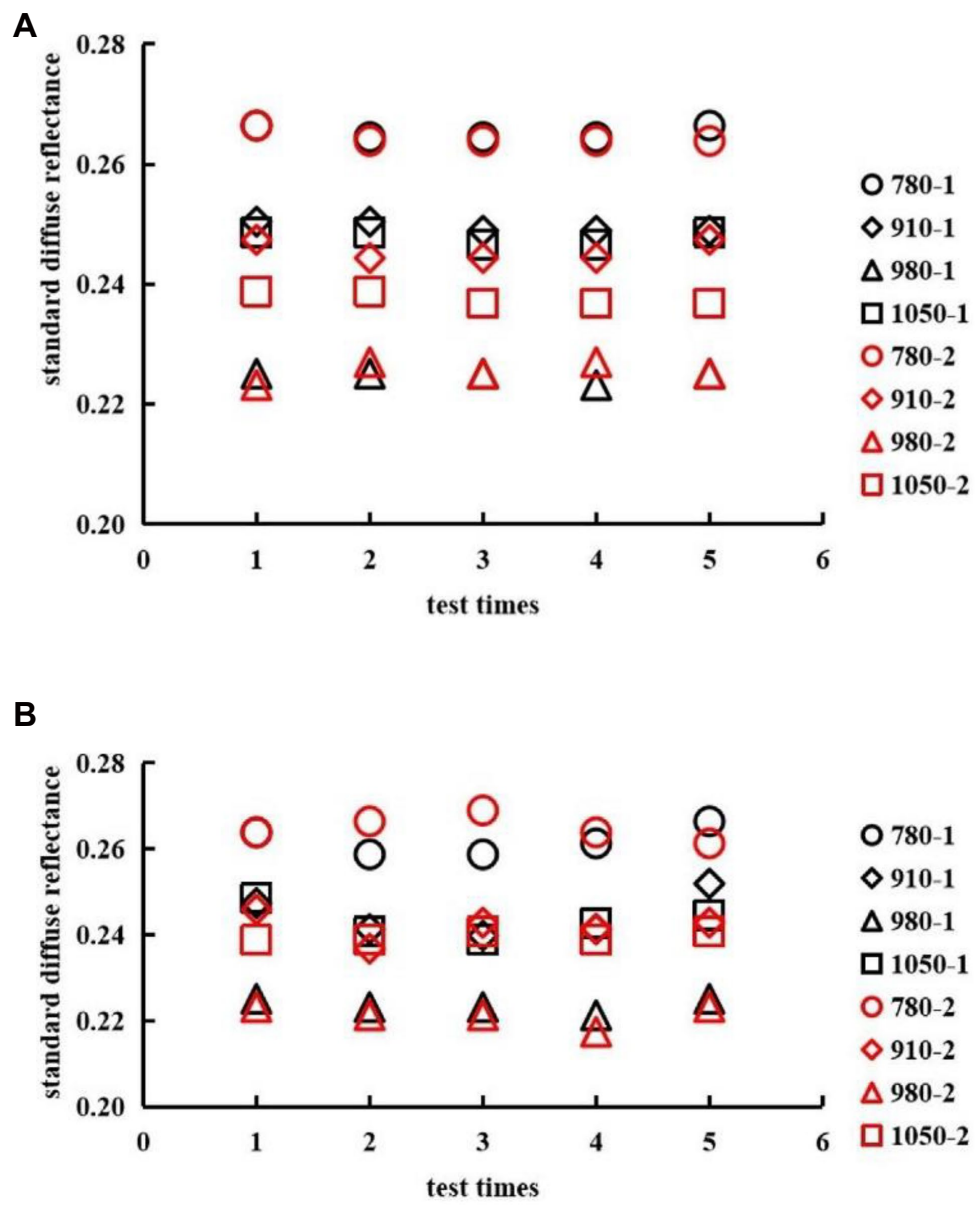
Performance test of the PSPMWG. **(A)** Noise level, **(B)** Repeatability.

The noise level and repeatability of the NIR PSPMWG are shown in Figure 17. Here, 780–1, 910–1, 980–1, and 1,050–1 refer to the RDR of sample #1 at the wavelengths of 780, 910, 980, and 1,050 nm, respectively. 780–2, 910–2, 980–2, and 1,050–2 are the RDR of sample #2 for the corresponding series of wavelengths. In the noise level test, the standard deviations of RDR of sample #1 were 0.11, 0.08, 0.09, and 0.11%. The standard deviations of RDR of sample #2 were 0.11, 0.17, 0.16, and 0.11%. In the repeatability test, the standard deviations of RDR of sample #1 were 0.33, 0.51, 0.16, and 0.38%. The standard deviations of RDR of sample #2 were 0.29, 0.33, 0.23, and 0.11%. In summary, the NIR PSPMWG can accurately collect the RDR of wheat grains.

#### Calibration Test

**Figure 18:**
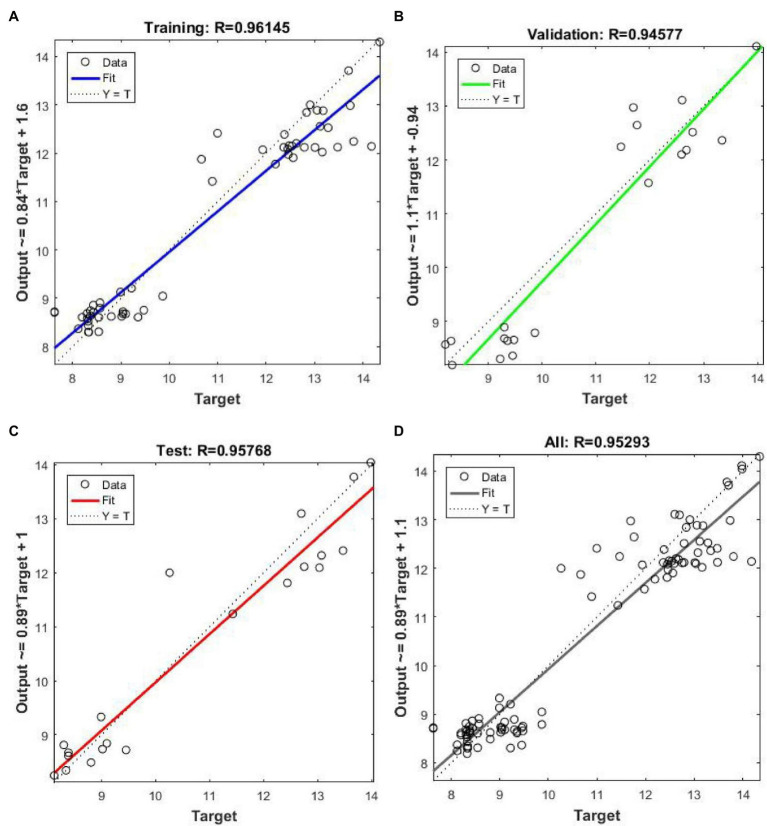
Scatter plot of the detection values and the physicochemical values. **(A)** Training set, **(B)** Validation set, **(C)** Test set, **(D)** All values.

The relationship between the detection values obtained by the NIR PSPMWG through the quality detection model and the physicochemical values is shown in Figure 18. In the figure, Target represents the physicochemical values of protein and moisture, Output represents the detection values. In the prediction of protein, the RMSE of the Training set was 0.7981% and MAE was 0.6060%; the RMSE of Validation set was 0.4218% and MAE% was 0.6229; the RMSE of the Test set was 0.4866% and MAE was 0.6515%. In the prediction of moisture, the RMSE of the Training set was 0.4598% and MAE was 0.3634%; the RMSE of Validation set was 0.4295% and MAE was 0.6468%; the RMSE of the Test set was 0.2161% and MAE was 0.3078%. Thus, the established quality detection model can effectively detect the moisture and protein content of wheat grains. The NIR PSPMWG can obtain the moisture and protein content of wheat grains in real-time.

## Discussion

The NIR PSPMWG based on NIR spectroscopy can quickly, efficiently, and nondestructively obtain grain quality information. Currently, full-band NIR commercial instruments such as NIR Systems 5,000 spectrometer and FOSS Infratec 1,241 spectrometer have achieved good results in the application of grain quality detection (Bao et al., 2010; Hexiao et al., 2011; Lin et al., 2014; Chen et al., 2019). Liu et al. (Hexiao et al., 2011) established a neural network model for wheat protein detection. The MSE of the calculated values and actual values of wheat protein content ranged from 0.799 to 0.09, and the average MSE of 0.178 transformed into RMSE of 0.4219%. This result is similar to the detection effect of our phenotypic sensors. However, these instruments are complex, bulky, non-portable, and expensive. Moreover, since only spectrum information was collected, and there was no quality detection model, these devices cannot be used for real-time on-site detection of grain quality. Given the above problems, in this study, we developed a NIR PSPMWG. Compared with existing full-band commercial instruments, the phenotypic sensor uses four wavelengths that are sensitive to grain quality and an optical system with a multi-source circular structure. The dimensions of the optical system were greatly reduced while ensuring that the diffuse reflectance spectrum of multi-grain wheat was collected. In existing commercial spectrometers, one needs to customize the function or manually calculate the quality information through the collected spectral information to realize grain quality detection the customization is costly and the manual calculation leads to poor real-time performance; thus, it is difficult to implement these instruments in actual applications. However, by collecting the RDR of wheat grain using the designed sensor, and coupling it with the quality detection model, the grain quality information was obtained in real-time.

Due to the different sensitive wavelengths of different grain quality components, the PSPMWG needs to change the wavelength of its light source for specific detection objects. In addition, the shape, size, and size distribution of different varieties of wheat grains affect the scattering coefficient, which can lead to measurement error in the RDR. Further studies are needed to explore the methods that can reduce the difference of scattering coefficient and improve the accuracy of RDR and ultimately the detection accuracy of the PSPMWG.

## Conclusion

1. A NIR PSPMWG was developed. An optical system with a multi-source circular structure was used to avoid the difference of incident light spots of multiple light sources. The light source drive circuit based on optical power feedback and the weak optical signal conditioning spectral acquisition circuit was designed to improve the luminous stability and signal-to-noise ratio of the sensor. The standard deviation of RDR at four wavelengths was less than 0.17 and 0.51% in noise level and repeatability test, indicating that the sensor developed in this study can accurately collect RDR of wheat grain.
2. The quality detection model of wheat grains was established. The RDR of wheat grains was collected by the phenotypic sensor, and the physicochemical values of moisture and protein were obtained by physicochemical analyses. In the Test set, the RMSEs of protein and moisture content of wheat were 0.4866 and 0.2161%, the MAEs were 0.6515 and 0.3078%, indicating that the phenotypic sensor can achieve real-time, on-site acquisition of RDR and quality information of wheat grains.

## Data Availability Statement

The raw data supporting the conclusions of this article will be made available by the authors, without undue reservation.

## Author Contributions

YL: data curation, visualization, writing—original draft. YL and DL: formal analysis. JN: funding acquisition and project administration. YL, DL, and HL: investigation. JN and YL: methodology and writing—review and editing. WC and YZ: resources. YL and XJ: software and supervision. XJ, YL, and HL: validation. All authors have read and agreed to the published version of the manuscript. All authors contributed to the article and approved the submitted version.

## Funding

This work was supported by the National Natural Science Foundation of China [grant number 31871524]; the Primary Research & Development Plan of Jiangsu Province of China [grant numbers BE2021308, BE2021304]; Six Talent Peaks Project in Jiangsu Province [grant number XYDXX-049]; and the 111 Project [grant number B16026].

## Conflict of Interest

The authors declare that the research was conducted in the absence of any commercial or financial relationships that could be construed as a potential conflict of interest.

## Publisher’s Note

All claims expressed in this article are solely those of the authors and do not necessarily represent those of their affiliated organizations, or those of the publisher, the editors and the reviewers. Any product that may be evaluated in this article, or claim that may be made by its manufacturer, is not guaranteed or endorsed by the publisher.

## Abbreviations

PSPMWG: phenotypic sensor about protein and moisture in wheat grain
RDR: relative diffuse reflectance
